# Multiple causes of an unexpected malaria outbreak in a high-transmission area in Madagascar

**DOI:** 10.1186/s12936-016-1113-0

**Published:** 2016-02-02

**Authors:** Thomas Kesteman, Solofoniaina A. Rafalimanantsoa, Harimahefa Razafimandimby, Heriniaina H. Rasamimanana, Vaomalala Raharimanga, Benjamin Ramarosandratana, Arsene Ratsimbasoa, Jocelyn Ratovonjato, Nohal Elissa, Laurence Randrianasolo, Alyssa Finlay, Christophe Rogier, Milijaona Randrianarivelojosia

**Affiliations:** Institut Pasteur de Madagascar, Avaradoha, BP 1274, 101 Antananarivo, Madagascar; Unité de recherche sur les maladies infectieuses et tropicales émergentes (URMITE)-UMR 6236, 27 boulevard Jean Moulin, 13385 Marseille Cedex 05, France; Fondation Mérieux, 17 rue Bourgelat, 69002 Lyon, France; Direction de Veille Sanitaire et de Surveillance Epidémiologique, Ministère de la Santé, Antananarivo, Madagascar; Direction de Lutte contre le Paludisme, Ministère de la Santé Publique, Antananarivo, Madagascar; US Centers for Disease Control and Prevention, Atlanta, GA USA; Institute for Biomedical Research of the French Armed Forces (IRBA), BP 73, 91223 Brétigny-Sur-Orge Cedex, France

**Keywords:** Malaria, Madagascar, Infectious disease outbreaks, Epidemiology

## Abstract

**Background:**

The malaria burden in Madagascar dropped down last decade, largely due to scale-up of control measures. Nevertheless, a significant rise of malaria cases occurred in 2011–2012 in two regions of the rainy South-Eastern Madagascar, where malaria is considered as mesoendemic and the population is supposed to be protected by its acquired immunity against *Plasmodium*. A multidisciplinary investigation was conducted in order to identify the causes of the outbreak.

**Methods:**

In March 2012, a cross-sectional study was conducted in 20 randomly selected clusters, involving the rapid diagnostic testing of all ≥6 month-old members of households and a questionnaire about socio-demographic data and exposure to malaria control interventions. Changes in environmental conditions were evaluated by qualitative interview of local authorities, climatic conditions were evaluated by remote-sensing, and stock outs of malaria supplies in health facilities were evaluated by quantitative means. Two long-lasting insecticidal nets (LLINs) were sampled in each cluster in order to evaluate their condition and the remanence of their insecticidal activity. The entomological investigation also encompassed the collection *Anopheles* vectors in two sites, and the measure of their sensitivity to deltamethrin.

**Results:**

The cross-sectional survey included 1615 members of 440 households. The mean *Plasmodium* infection rate was 25.6 % and the mean bed net use on the day before survey was 71.1 %. The prevalence of *Plasmodium* infections was higher in 6–14 year-old children (odds ratio (OR) 7.73 [95 % CI 3.58–16.68]), in rural areas (OR 6.25 [4.46–8.76]), in poorest socio-economic tercile (OR 1.54 [1.13–2.08]), and it was lower in individuals sleeping regularly under the bed net (OR 0.51 [0.32–0.82]). Stock outs of anti-malarial drugs in the last 6 months have been reported in two third of health facilities. Rainfalls were increased as compared with the three previous rainy seasons. Vectors collected were sensitive to pyrethroids. Two years after distribution, nearly all LLINs collected showed a loss of physical integrity and insecticide activity,

**Conclusions:**

Increased rainfall, decreasing use and reduced insecticide activity of long-lasting insecticide-treated nets, and drug shortages may have been responsible for, or contributed to, the outbreak observed in South-Eastern Madagascar in 2011–2012. Control interventions for malaria elimination must be sustained at the risk of triggering harmful epidemics, even in zones of high transmission.

## Background

**Fig. 1:**
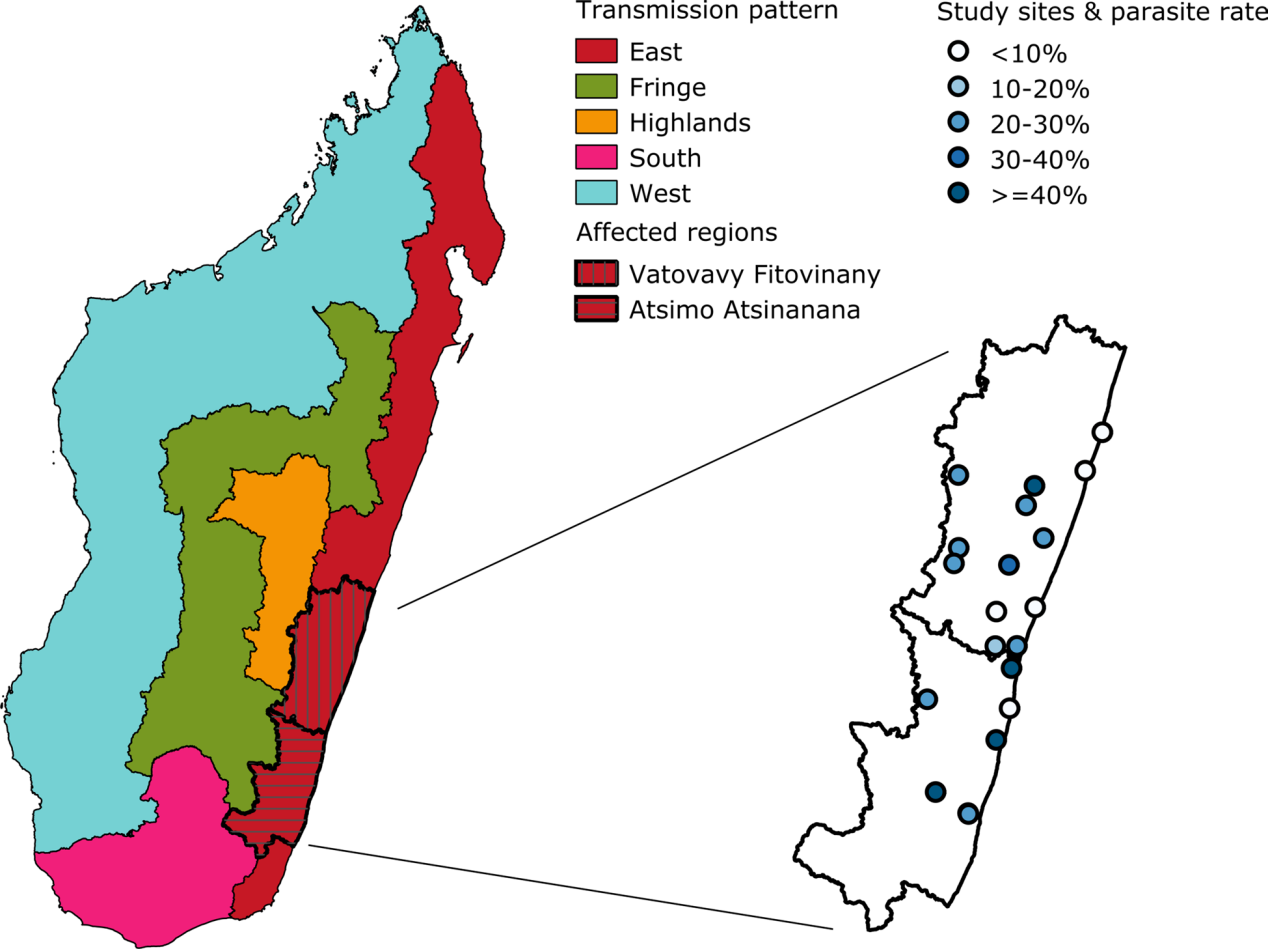
Malaria transmission patterns of Madagascar, location of the regions affected by the outbreak, and study sites and their parasite rates

The rainy Eastern region of Madagascar is an area of endemic and perennial malaria transmission. It had been a malaria hyperendemic area prior to 2006 with annual entomological inoculation rate ranging from 16 to 240 infective bites/person and a *Plasmodium falciparum* parasite rate up to 50 % among children [1–4]. The malaria burden in Madagascar has dropped from 2006 levels following the large scale-up of malaria control measures. After the massive scale-up of malaria control interventions during the years 2000’s, malaria endemicity of the Eastern transmission pattern shifted from hyperendemic to mesoendemic, with a parasite rate among children under-five years old of 16.4 % in 2011 [5, 6]. Given the historically high levels of malaria transmission, the population was thought to be protected by its acquired immunity against *Plasmodium* infection and this area was not considered a zone prone to malaria epidemics [1, 3]. No malaria outbreaks have previously been described in this geographic zone of Madagascar [4]. In February of 2012, an increase in malaria cases was reported in Vatovavy Fitovinany and Atsimo Atsinanana regions (Fig. 1) through the fever sentinel surveillance system [7]. Biologically confirmed clinical malaria cases increased almost threefold when compared to the same period in 2010–2011. The rise in cases began in December 2011 [5]. A multidisciplinary investigation, involving epidemiological and entomological methods, was conducted to identify factors that could have caused the malaria outbreak.

## Methods

### Epidemiological studies

In March 2012, in collaboration with the Ministry of Health, a cross-sectional survey was carried out in 20 *fokontany* or villages (clusters) selected by probability sampling (proportional to population size) among the *fokontany* of Vatovavy Fitovinany and Atsimo Atsinanana regions. In each cluster, households were selected at random so as to include 80 household members per cluster interviewed with informed consent. The questionnaire was administered to each household member and included questions on socio-demographic status, history of exposure to malaria prevention and control measures, and travel during the preceding 3 months. A rapid diagnostic test for malaria (RDT) was performed on all household members aged 6 months or older. The CareStart^®^ Malaria RDT (Access Bio Inc., Monmouth Junction (NJ), USA) was used, since it is the RDT commonly used in the public health system in Madagascar. Individuals with a positive RDT result were treated with artemisinin-based combination therapy (ACT) according to national guidelines. Household socioeconomic terciles were created by principal component analysis of socioeconomic variables [8]. Generalized estimating equation logistic regression models were constructed to assess relationships between possible risk factors and parasitemia, accounting for clustering.

In each selected village the nearest health care facilities were visited to assess the availability of ACT and RDTs from September 2011 to February 2012, using a standardized quantitative questionnaire. Rainfall and temperature data collected by remote climatic sensing during the 2011–2012 rainy season and the previous 3 years were retrieved from databases available on the internet [9] and compared. Qualitative interviews of health workers, health authorities and administrative authorities have been conducted in order to assess recent changes in socio-economic conditions, agricultural habits, environmental conditions, and movements of populations.

Given the emergency and to avoid any delay, upon request of the Ministry of Health of Madagascar, the study protocol was not submitted to the National Ethic Committee. Nevertheless, the investigators’ practices were fully in line with the ethical principles according to the Helsinki Declaration. Particularly, informed consent was obtained from the individuals or the parents/tutors of the children before inclusion and all individual data remained confidential.

### Entomological studies

*Anopheles* vectors, adults and larvae, were collected at two sites in the study area during the same period. Adult vectors were collected by human landing catches involving approximately five catchers indoors and five outdoors, during two nights in the first site and one night in the second site. Vector species was determined morphologically. The infection of vectors with *Plasmodium* and the origin of blood meals were determined by ELISA. Sensitivity of wild caught female adults to deltamethrin 0.05 % was determined. The feeding behaviour (endophagic vs exophagic) of female adults was also studied. Two long-lasting insecticidal nets (LLINs) per cluster were randomly sampled during the cross-sectional survey to evaluate physical integrity and insecticidal activity (bioefficacy) using methods recommended by the World Health Organization (WHO) [10, 11].

## Results

### Epidemiological studies

Among the 20 clusters there were 1615 individuals living in 440 households, owning a total of 692 bed nets (BNs). Data were collected from 1531 (94.8 %) individuals. The *Plasmodium* infection rate was 25.6 % (95 % confidence interval (CI) 23.4–27.9 %, range among clusters 3.1–57.5 %, Table 1) and the reported BN use by individuals on the night before the survey was 71.1 % [95 % CI 68.8–73.4 %]. Among 589/692 (85.1 %) of BNs for which the net brand was clearly identified, 574 (97.4 %) were LLINs. Median age of LLINs was 2.3 years [interquartile range (IQR) 2.0–3.0 years]. Indoor residual spraying was not performed in this area.

**Table 1:** Bivariate and multivariate multilevel analysis of the association between *Plasmodium* infection (assessed by RDT) and demographic and epidemiological variables, South-Eastern Madagascar, 2012

Variable	N (% RDT positive)	Bivariate		Multivariate	
		Crude OR [95 % CI]	p	Adjusted OR [95 % CI]	p
Bed net use					
Every night	1009 (22.6)	0.49 (0.33–0.72)	<0.001	0.51 (0.32–0.82)	0.005
Irregular	225 (32.0)	0.84 (0.60–1.19)	0.331	0.95 (0.62–1.43)	0.791
Never	297 (31.0)	1.00		1.00	
Age group					
0–5 years	410 (32.9)	5.15 (2.38–11.15)	<0.001	5.51 (2.48–12.26)	<0.001
6–14 years	395 (39.2)	7.42 (3.52–15.65)	<0.001	7.73 (3.58–16.68)	<0.001
15–49 years	594 (15.7)	2.21 (1.06–4.63)	0.036	2.32 (1.09–4.96)	0.029
≥50 years	132 (6.8)	1.00		1.00	
Sex					
Male	630 (28.1)	1.00		1.00	
Female	901 (23.9)	0.79 (0.62–1.01)	0.064	0.97 (0.73–1.30)	0.862
Travel history					
None	1179 (27.0)	1.00			
≤10 km	217 (24.0)	0.77 (0.48–1.23)	0.271		
11–50 km	103 (16.5)	0.65 (0.37–1.14)	0.131		
>50 km	32 (15.6)	0.69 (0.25–1.91)	0.472		
Socio-economic tercile					
First (poorest)	566 (32.2)	1.74 (1.31–2.30)	<0.001	1.54 (1.13–2.08)	0.006
Second	459 (27.0)	1.48 (1.01–2.15)	0.043	1.46 (0.99–2.15)	0.058
Third (wealthiest)	506 (17.0)	1.00		1.00	
Area type					
Rural	1311 (29.0)	8.59 (6.45–11.44)	<0.001	6.25 (4.46–8.76)	<0.001
Suburban	153 (5.9)	1.26 (0.67–2.40)	0.475	1.15 (0.59–2.23)	0.677
Urban	67 (4.5)	1.00		1.00	
ACT shortages last 6 months					
No (4 sites)	272 (21.0)	1.00			
Yes (14 sites)	1131 (27.9)	1.46 (0.70–3.06)	0.313		
Unknown (2 sites)	128 (14.8)	0.79 (0.22–2.78)	0.711		

The prevalence of *Plasmodium* sp. infections was significantly higher among (i) individuals who did not sleep under a BN every night; (ii) 6–14 year-old children; (iii) residents of households with lower socioeconomic status; and (iv) in rural areas, both in bivariate and multivariate analysis (Table 1). There was no evidence of massive population movements in this area, and no correlation was observed between travel history and RDT positivity (Table 1). Nightly BN use was associated with lower parasite prevalence (OR 0.51 [95 % CI 0.32–0.81], Table 1).

Of the 47 health facilities that served the selected study site communities, 31 (66 %) experienced at least one stock-out of at least one dosage type of ACT since September 2011 (mean stock-out duration 51 days); and 14 (45 %) had stock-outs at the time of the survey. The interviews of health workers indicated that they adapted to shortages by adjusting paediatric doses for adults if adult doses were missing or vice versa. When analysed by cluster, stock-outs of ACT during the 6 months prior to the survey were associated in univariate analysis with a higher prevalence of *Plasmodium* infection (OR 1.46 [95 % CI 0.7–3.0], Table 1) although the association was not significant. Out of 31 health facilities supposed to have RDTs, 20 (64.5 %) experienced stock outs of RDTs since September 2011.

Rainfall during the 2011–2012 rainy season was higher than the previous three rainy seasons in the two regions; four districts had over 40 % more rainfall, two districts 30–39 % more rainfall, two districts 15–29 % more rainfall and one district had no change in rainfall (0.5 % more rainfall). No significant change in temperature was observed. Interviews with health and administrative authorities did not support changes in agriculture/husbandry habits or soil use.

### Entomological studies

Endo- and exophagic *Anopheles gambiae* (N = 96) and endophagic *An. mascarensis* (N = 70) were identified in two nights in the first site. Exophagic *An. mascarensis* (N = 170), a few *An. gambiae* (N = 6) and *An. funestus* (N = 5) were identified in the other site in one night. Species and feeding behaviour (including biting hours) of *Anopheles* vectors found were consistent with findings prior to LLIN mass distributions campaigns [12]. All vectors tested (59 *An. mascarensis* and 16 *An. gambiae*) were sensitive to deltamethrin. Only one vector was found to be infected by *Plasmodium*, but all vectors were found to have fed on humans.

Thirty-nine LLINs were collected from households belonging the 20 clusters investigated in the cross-sectional study. The median age of sampled LLINs was 2.3 years [IQR 2.0–3.0 years]. Physical integrity was “good” for six (15.0 %) LLINs, 17 (42.5 %) LLINs were “damaged” and 17 (42.5 %) were “too torn”. Cone bioassay results showed only one (2.6 %) LLIN achieving the desired knocked-down threshold of 95 % and no LLIN met the WHO efficacy requirement of 80 % mortality using a susceptible strain of *An.arabiensis* mosquitoes. This strain was previously characterized in the Institut Pasteur in Madagascar for insecticide susceptibility using standard WHO impregnated paper tests and 100 % mortality was observed with deltamethrin 0.05 %.

## Discussion

The present study identified several factors that may have caused, or exacerbated, the outbreak of malaria observed in these regions. First, the increase in rainfall likely triggered a rise in vector population, although this could not be confirmed by the entomological investigations. Indeed, the entomological inoculation rate could not be reliably calculated in the present study because only one vector was found infected by *Plasmodium*. Second, the intensive control interventions deployed in the previous decade could have weakened the population’s immunity against clinical malaria. The meteorological conditions resulting in an increased *Plasmodium* transmission thus translated into an increase in the number of clinical cases of malaria.

Third, less intensive control interventions made possible for the parasite to keep circulating and the number of cases to keep growing. The individual BN use was 71.1 % [95 % CI 68.8–73.4 %] in this study, which is lower compared to results from the Malaria Indicator Survey in 2011 (88.6 % in the Eastern transmission pattern [95 % CI 88.0–89.2 %]) [6]. Nightly BN use was significantly associated with lower parasite prevalence (OR 0.51 [95 % CI 0.32–0.81], Table 1). This association was comparable to reports from other sub-Saharan African settings, including a meta-analysis where the overall OR of sleeping under an insecticide-treated net was 0.76 [95 % CI 0.58–0.99] [13]. Nonetheless, the bio-efficacy of a subsample of LLINs was found to be poor after 2 years while the expected survival was at least 3 years. This may have impaired the overall protective effect of the LLINs at community level provided by the reduction of endophagic and endophilic vectors, and contributed to the observed increase in malaria cases. Finally, the results show that an important proportion of health facilities experienced stock-outs in ACT and/or RDTs. Even if health workers adapted the dosages not to leave infected individuals untreated, it is likely that treatments were not optimal during stock-outs. Part of these shortages preceded the outbreak and may have precluded the treatment of *Plasmodium* infections, consequently increasing the parasite reservoir. The causal relationship between shortages and increase in malaria cases could not be established from the present study and it’s of course likely that stock outs were the consequence of the unanticipated increase in the number of cases to be treated. Overall, whatever was the *princeps movens*, inappropriate case management may have aggravated the outbreak.

Other possible causes or favouring factors have been discarded. Entomological investigations showed that no major change in vectors’ biting behaviour or species had occurred. Qualitative interviews evidenced neither changes in agriculture or husbandry practices–that could have created new breeding sites for vectors–, nor population movements between the regions studied and regions with lower transmission.

The main limitation of the present study is that some factors that may have played a role in the outbreak were not evaluated. The sensitivity of local parasite strains for ACT was not verified. Nevertheless, reduced sensitivity of *Plasmodium falciparum* to artemisinin was not detected in recent clinical [14] or molecular [15] surveys in Madagascar. More importantly, changes in socio-economic and wealth conditions were not investigated. The political and economic crisis that lasts since 2009 has seriously affected the population of Madagascar and impacts their health, in particular through a reduction in healthcare workforce, that is especially marked in rural areas [16]. It is likely that this adverse context has played a role in the emergence or the expansion of the outbreak of 2012.

Overall these results emphasize the importance of maintaining a constant- or increasing- level of malaria control. This corroborates the recent recommendation of the Global Malaria Programme not to scale back malaria control interventions before transmission has been halted and surveillance/response capacities have been developed [17].

## Conclusions

Multiple factors including increased rainfall, waning BN use, poor physical quality and bioefficacy of LLINs, and ACT shortages may have been responsible for, or contributed to, the malaria outbreak observed in South-Eastern Madagascar in 2011–2012. A decline in malaria endemicity following large-scale prevention and control measures can result in a population with decreased malaria immunity rendering them vulnerable to malaria outbreaks after only a few years. The sustainability of effective malaria prevention and control measures is imperative in the path from malaria control to elimination.

## Abbreviations

ACT: artemisinin-based combination therapy
BN: bed net
CI: confidence interval
IQR: interquartile range
LLIN: long-lasting insecticidal net
OR: odds Ratio
RDT: rapid diagnostic test
WHO: World Health Organization

## References

[1] Hay SI, Smith DL, Snow RW (2008). Measuring malaria endemicity from intense to interrupted transmission. Lancet Infect Dis.

[2] Hay S, Guerra C, Tatem A (2004). The global distribution and population at risk of malaria: past, present, and future. Lancet Infect Dis.

[3] Mouchet J, Blanchy S (1995). [Particularities and stratification of malaria in Madagascar] (in French). Cah Santé.

[4] Mouchet J, Carnevale P, Coosemans M, Julvez J, Manguin S, Richard-Lenoble D (2004). Biodiversité du paludisme dans le monde.

[5] Roll Back Malaria: Focus on Madagascar. Progress and Impact Series-Country Reports. Geneva; 2013.

[6] Institut National de la Statistique, Programme National de Lutte contre le Paludisme, ICF International: Enquête sur les indicateurs du paludisme à madagascar (EIPMD). Antananarivo, Madagascar; 2012.

[7] Randrianasolo L, Raoelina Y, Ratsitorahina M, Ravolomanana L, Andriamandimby S, Heraud J-M (2010). Sentinel surveillance system for early outbreak detection in Madagascar. BMC Public Health.

[8] Filmer D, Pritchett LH (2001). Estimating wealth effects without expenditure data—or tears: an application to educational enrollments in states of India. Demography.

[9] US CPC Gauge-Based Analysis of Global Daily Precipitation [http://iridl.ldeo.columbia.edu/SOURCES/.NOAA/.NCEP/.CPC/.UNIFIED_PRCP/.GAUGE_BASED/.GLOBAL/.v1p0/.REALTIME/.rain/].

[10] World Health Organization: Guidelines for monitoring the durability of long-lasting insecticidal mosquito nets under operational conditions. 2011.

[11] WHO Vector Control Technical Expert Group: Estimating functional survival of long-lasting insecticidal nets from field data contents. Geneva; 2013.

[12] Andrianaivolambo L, Domarle O, Randrianarivelojosia M, Ratovonjato J, Le Goff G, Talman A (2010). Anthropophilic mosquitoes and malaria transmission in the eastern foothills of the central highlands of Madagascar. Acta Trop.

[13] Lim SS, Fullman N, Stokes A, Ravishankar N, Masiye F, Murray CJL (2011). Net benefits: a multicountry analysis of observational data examining associations between insecticide-treated mosquito nets and health outcomes. PLoS Med.

[14] Zwang J, Olliaro P, Barennes H, Bonnet M, Brasseur P, Bukirwa H (2009). Efficacy of artesunate-amodiaquine for treating uncomplicated *falciparum* malaria in sub-Saharan Africa: a multi-centre analysis. Malar J.

[15] Kamau E, Campino S, Amenga-Etego L, Drury E, Ishengoma D, Johnson K (2015). K13-propeller polymorphisms in *Plasmodium falciparum* parasites from sub-Saharan Africa. J Infect Dis.

[16] Barmania S (2013). Madagascar’s health challenges. Lancet.

[17] WHO Global Malaria Programme: Risks associated with scale-back of vector control after malaria transmission has been reduced. Geneva; 2015.

